# Proteomic analysis of Exosomes derived from the Aqueous Humor of Myopia Patients

**DOI:** 10.7150/ijms.51735

**Published:** 2021-03-10

**Authors:** Ching-Yao Tsai, Chueh-Tan Chen, Chin-Hui Lin, Chen-Chung Liao, Kate Hua, Chung-Hua Hsu, Chian-Feng Chen

**Affiliations:** 1 Department of Ophthalmology, Taipei City Hospital, Taipei, Taiwan.; 2 MS Program in Transdisciplinary Long Term Care, Fu Jen Catholic University, New Taipei City, Taiwan.; 3 Community Medicine Research Center and Institution of Public Health, National Yang Ming Chiao Tung University, Taipei, Taiwan.; 4 Institute of Traditional Medicine, National Yang Ming Chiao Tung University, Taipei, Taiwan.; 5 Cancer Progression Research Center, National Yang Ming Chiao Tung University, Taipei, Taiwan.; 6 Metabolomics-Proteomics Research Center, National Yang Ming Chiao Tung University, Taipei, Taiwan.; 7 Department of Chinese Medicine, Taipei City Hospital, Linsen, Chinese Medicine, and Kunming Branch, Taipei, Taiwan.

**Keywords:** aqueous humor, exosome, myopia

## Abstract

**Objectives:** Myopia is the most common refractive vision disorder. In recent years, several studies have suggested that the alteration of the exosomal protein levels in the aqueous humor (AH) is associated with the development of several eye diseases. Therefore, we aimed to explore the exosomal protein profile of the AH from myopia patients.

**Methods:** Exosomes were isolated from the AH. The quality, concentration, and size distribution of exosomes for each patient were measured using nanoparticle tracking analysis system. Then, the exosomal proteins were purified and digested by trypsin for liquid chromatography-tandem mass spectrometry (LC-MS/MS) analysis.

**Results:** There was no significant difference observed between the myopia and control when comparing the concentration and size distribution of exosomes in the AH for each sample. Based on LC-MS/MS analysis, myopia patients had higher and more complex exosomal peptide content. We found two proteins that were common in AH exosomes and eight proteins that were highly expressed in the myopia group.

**Conclusions:** Our results provide pioneering findings for the exploration of the exosomal protein profile in myopia development. Further studies may provide significant information for the diagnosis, clinical treatment, and prognosis of myopia.

## Introduction

Myopia is the most common refractive vision disorder where light focuses in front of the retina 1. The severity of myopia is usually measured in diopters: low, -3.00 diopters or less; moderate, -3.00 to -6.00 diopters; and high, more than -6.00 diopters 2. Genetic and environmental factors have been shown to influence the occurrence and development of myopia, but the detailed mechanism is still largely unknown 3. Myopia is known as a milder disease for the optical error can be corrected by wearing glasses, contact lenses, or refractive surgery 2, 4. However, it is becoming an ever-increasing global health concern as the prevalence of myopia has risen sharply in the past 50 years 5. In Taiwan, the prevalence and severity of myopia among the younger generation have increased rapidly over the past two decades 6. The rupture rate is as high as 70.3% among individuals 12-19 years old but only 5.6% among individuals older than 65 years 7. With a higher degree of myopia, there is greater risk for complications such as macular degeneration, retinal detachment, cataracts, and glaucoma 8. Myopia prevention and treatment is an urgent public health issue in Taiwan.

The aqueous humor (AH) is the clear liquid filling in the anterior and posterior chamber of the eye 9. It is secreted by the pigment-free epithelial cells of the ciliary body and serves for many important functions 9. First, the dynamic balance of AH production and discharge is important to maintain the shape and optical properties of the eye 10. AH fluid is also responsible for supplying nutrients and removing cellular waste for intraocular tissues 9, 10. Recently, several studies have suggested that the alteration of protein levels in AH associated with the development of several eye diseases 11-16. For example, the severity of corneal edema in eyes with bullous keratopathy has been associated with the levels of specific cytokines in the AH 17. Elevated TGFβ2 (transforming growth factor β2) levels in AH of cataract patients is a potential risk factor for capsular contraction syndrome 18, 19. Therefore, studying the composition of the AH will help to understand the process of disease development and to explore the clinical treatments.

Exosomes are composed of a lipid bilayer containing transmembrane proteins, enclosing cytosolic proteins, and RNAs 20, 21. They are secreted from cells and are released into body fluids (i.e., blood, urine, tears, and spinal fluid), which can be transported between adjacent or distant cells via the circulatory system 21. They are considered as important messengers for cell-to-cell communication 21. An increasing number of studies have reported that proteins or miRNAs in exosomes derived from the AH may be associated with ophthalmic diseases 22-25. For example, trabecular meshwork cells released exosomes containing myocilin under the stimulation of the external environment 26, 27. Different expression profiles of exosomal miRNAs were found to be associated with glaucoma and myopia 28, 29. To analyze the alteration of exosomal proteins that occur with myopia, we aimed to compare the individual protein profile from nine high myopia patients with nine control patients by using liquid chromatography-tandem mass spectrometry (LC-MS/MS).

## Materials and Methods

### AH sample collection

The study protocol was approved by the Research Ethics Committee of Taipei City Hospital (TCHIRB-10604109) and conducted according to the tenets of the Declaration of Helsinki. All study participants provided written informed consent before their enrollment. Human AH samples were collected from patients undergoing cataract surgery at the Zhongxing branch of Taipei City Hospital. The diagnostic criterion for myopia was defined as axial length of more than 26 mm. The control eyes were collected from senile cataract patients who were free from other ocular or systemic diseases. Approximately 100 μL of AH was collected from each patient by anterior chamber paracentesis, using a needle inserted through the peripheral cornea at the beginning of the procedure. Undiluted AH samples were collected in sterile tubes and stored at -80 °C until further study.

### Isolation of Exosomes from the AH

Exosomes were isolated from the AH using the ExoQuick precipitation solution (System Biosciences, Inc., Mountain View, CA) according to its recommended procedure. Approximately 100 μl of AH was centrifuged at 3000  ×  g for 15 minutes to remove cellular debris, after which the supernatants were collected. Phosphate-buffered saline (PBS) buffer was added to the supernatants to achieve a final volume of 250 μl. Then, 63 μl of precipitation solution was added to the supernatants and incubated overnight. The exosome pellets were collected by centrifugation at 12,000  ×  g for 90 minutes and then suspended in 100 μl PBS. The concentration and size distribution of the vesicles were measured by NanoSight NS10 (Malvern Instruments, Rancho Cucamonga, CA).

### Proteins purification and LC-MS/MS analysis

Exosomes were lysed by radioimmunoprecipitation assay (RIPA) buffer to extract the exosomal proteins. Then, the proteins were digested by trypsin (SMART Digest Trypsin Kit, Thermo Fisher Scientific; Waltham, MA), desalted (Millipore® Ziptips Micro-C18; Sigma-Aldrich, Milwaukee, WI), purified (SOLAµ™ SPE Plates; Thermo Fisher Scientific; Waltham, MA), and dissolved in 0.1% formic acid for LC-MS/MS analysis (LTQ Orbitrap Velos, Thermo Scientifics; Waltham, MA) (service provided by the Mass Core Facility of Genomics Research Center, Academia Sinica). The acquired proteomic raw data files were then applied to search against a UniProt human protein database by using PEAKS Studio 7.5 (Bioinformatics Solutions, Waterloo, Ontario, Canada). The settings in PEAKS Studio 7.5 combined with UniProt for searching the protein database were as follows: enzyme set as trypsin with a maximum of two missed cleavage site precursor and fragment mass tolerance of 20 ppm and 0.8 Da, respectively. Finally, spectral counts obtained from each peptide were normalized to the total spectral counts recorded for all peptides in a sample.

### Statistical analyses

Data are expressed as the mean ± standard error of the mean. The measurements of axial lengths and exosome concentration were analyzed using a paired sample t-test. All data analysis was performed using SPSS version 24 (SPSS, Inc; Chicago, IL). A P value of less than 0.05 was considered to indicate a statistically significant difference. The principal component analysis (PCA) plot of all samples was generated using Partek Genomics Suite 7.18 (Partek Inc., St. Louis, MO).

## Results

### Samples collection and exosome isolation

**Figure 1:**
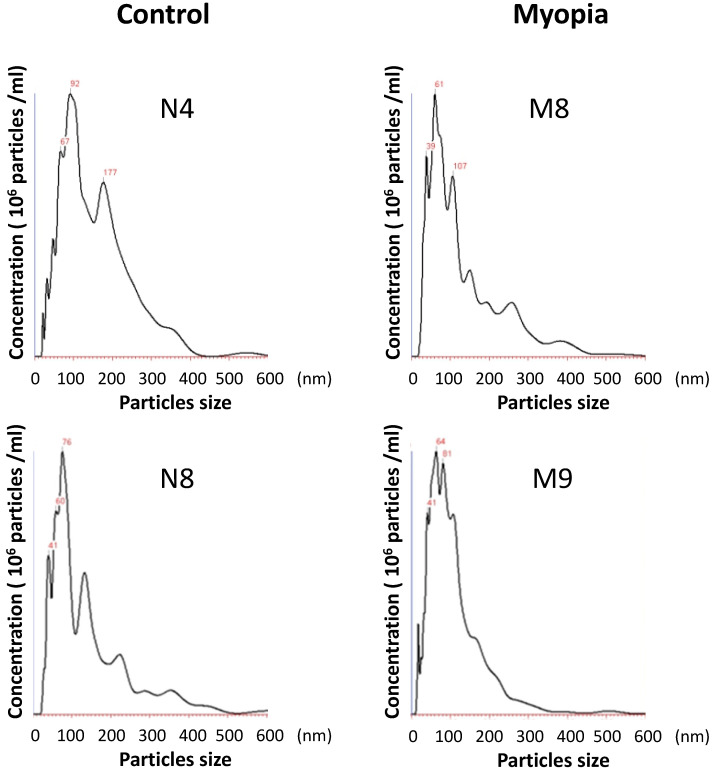
** The size distribution of AH exosomes.** Representative nanoparticle tracking analysis of exosomes isolated from the AH sample of patients with myopia (M) and controls (N).

The AH samples were collected from 18 patients undergoing cataract surgery at Taipei City Hospital. The patients were assigned to the myopia group if their axial length was more than 26 mm. There were no significant differences in the clinical characteristics between myopia and control group, except the axial length (Table 1). Exosomes were isolated from the AH by using exosome purification kit for each individual patient. The concentration and the size distribution of the purified small vesicles were determined by nanoparticle tracking analysis system. Calculating the diameter of these small vesicles, most were found to be in the expected range of exosomes (30-150 nm) (Fig. 1). Comparing the concentration of exosomes in AH for each sample, there was no significant difference observed between myopia and control groups (Table 2).

### Exosomal protein isolation and LC-MS/MS analysis

**Figure 2:**
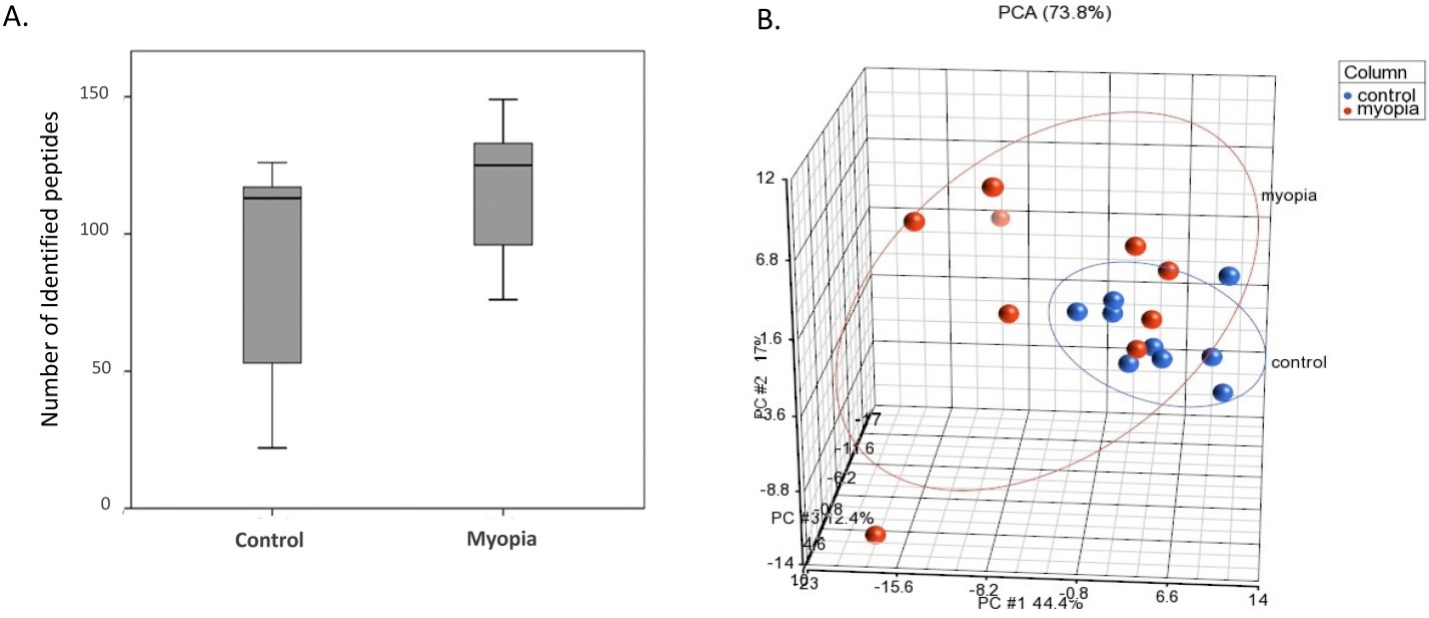
** LC-MS/MS-identified peptides from myopia and control groups.** A) Box plot of identified peptides from individual myopia and control patients. B) The PCA plot of all samples was generated to assess the variability of peptide expression in myopia and control patients. (red, myopia; blue, control).

Proteins isolated from the exosomes were purified and digested by trypsin for LC-MS/MS quantitation. As shown in Figure 2A, the number of peptides identified from the myopia group was more than that in the control group. PCA was applied to further demonstrate similarities and differences in the exosomal peptides content. On a PCA plot, samples with similar peptide profiles can be positioned in proximity to each other. The position of each sample was plotted against the PC1, PC2, and PC3 axes in a three-dimensional space. The PCA resulted in the delineated of two distinct positions in the three-dimensional space for myopia and control groups (Fig. 2B). In addition, the myopia group presented more diversity than the control group (Fig. 2B).

### Potential protein makers for AH exosomes

**Figure 3:**
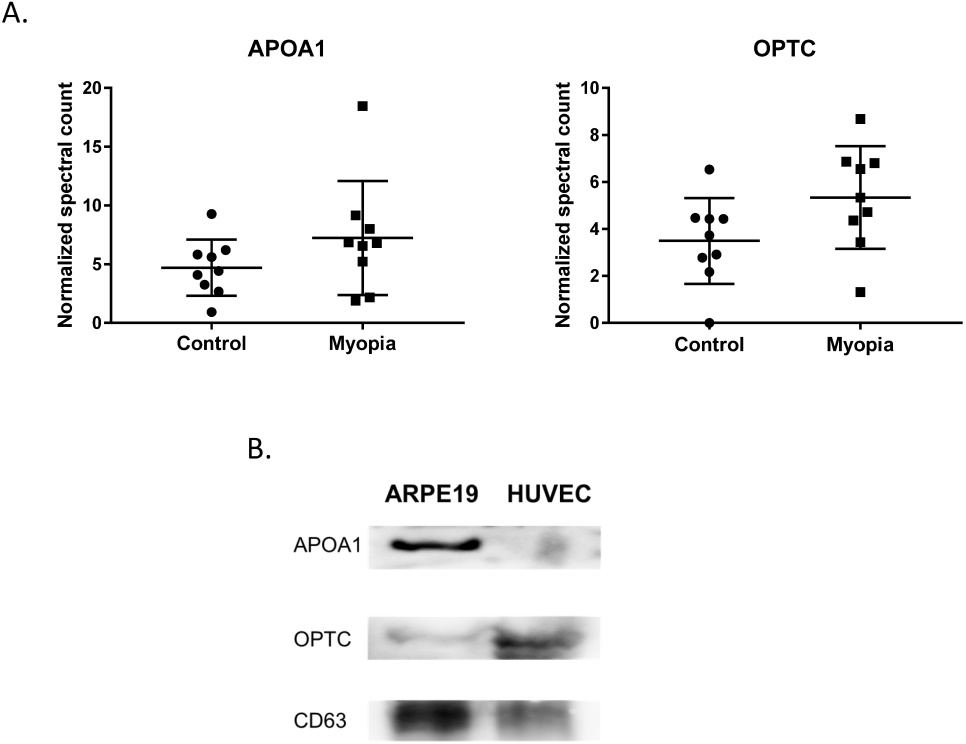
** Common proteins in AH exosome.** A) Normalized spectral count (NSC) of AH common proteins detected by LC-MS/MS. Scatterplot presenting the mean ± SD for the NSC of proteins detected in each patient from myopia or control. B) Exosomes proteins from ARPE19 (retinal pigmented epithelial cell) and HUVEC (umbilical vein endothelial cells) were analyzed by western blotting. CD63 is a well-known common exosome marker.

In summary, 164 peptides were identified from myopia and control exosomes. By searching the peptide sequences in the UniProt peptide database (www.uniprot.org), we obtained their protein names. Comparing our results with previous proteomic analysis of exosome from the AH, there were 15 proteins in our results have been reported in the studies of age-related macular degeneration patients 25. More interestingly, we found that two of them, apolipoprotein A1 (APOA1) and opticin (OPTC), commonly had high expression in either myopia or control patients (Fig. 3A). To confirm our finding, we next test their protein expression in exosome from retinal pigmented epithelium cell lines (ARPE19). Western blot results indicated both APOA1 and OPTC were present in exosomes (Fig. 3B). APOA1 protein has been found in exosomes derived from saliva and urine 30, 31, but there are no reports suggesting OPTC as an exosomal protein. Because OPTC expression is common in various eye tissues 32, we suggested that OPTC could be a potential AH-specific exosomal marker.

### Myopia specific exosomal proteins

**Figure 4:**
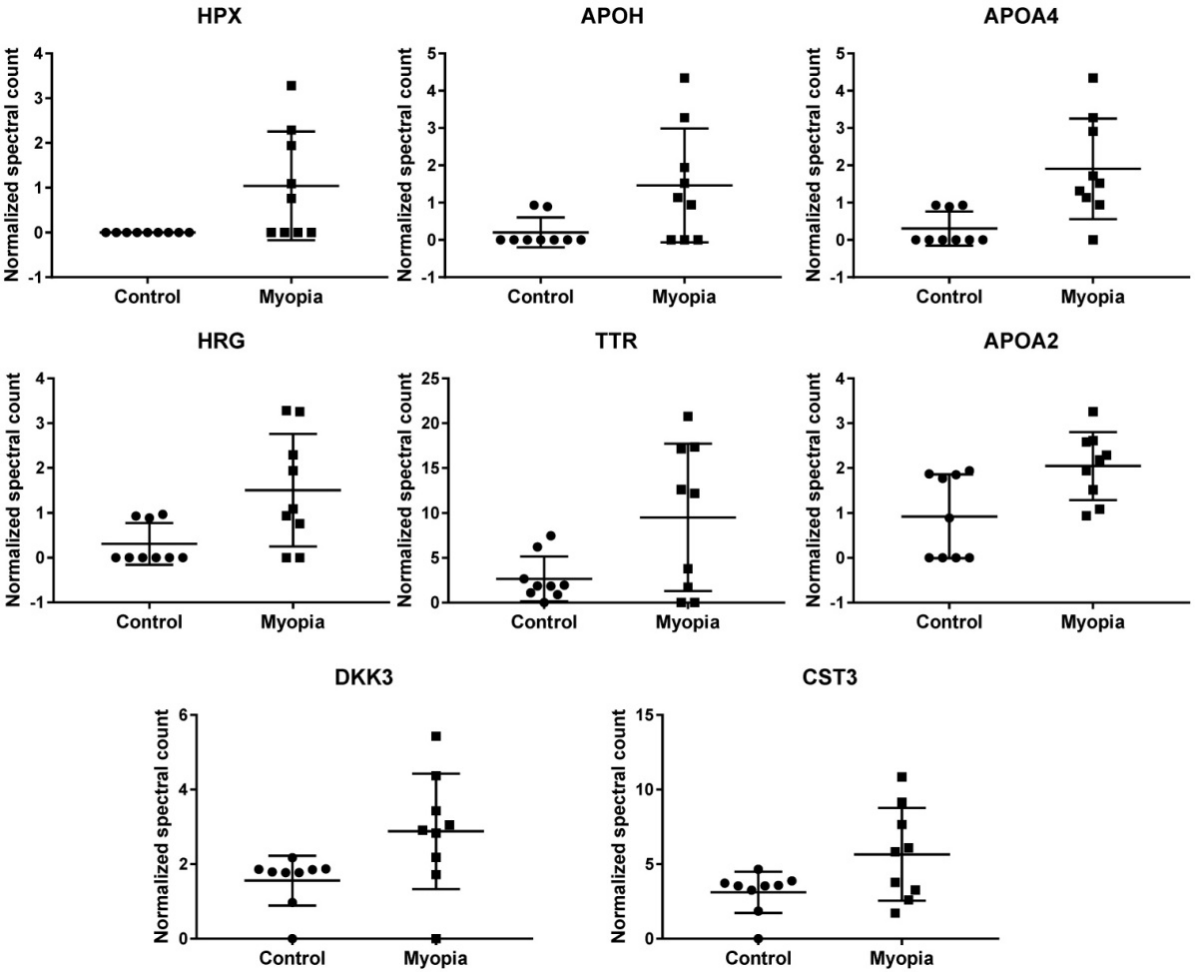
** Normalized spectral count (NSC) of myopia-specific proteins were detected from LC-MS/MS.** Scatterplot presenting the mean ± SD for the NSC of proteins detected in each patient from myopia or control. *P* values were calculated by paired two-sample *t* tests.

Comparing the exosomal peptides between myopia and control patients, 35 peptides were significantly different between myopia and control groups (t- test, *P* < 0.05) (Table 3). These peptides belong to 8 known (Fig. 4) and 17 unidentified proteins. More interestingly, all were significantly increased in myopia patients, suggesting that myopia patients secreted more exosomal proteins to the AH. In previous studies, transthyretin (TTR) and hemopexin (HPX) have been detected in the serum of high myopia patients 33, 34. In addition, TTR has also been found in the AH of high myopia patients 35. In summary, these results support our finding that these are myopia-specific exosomal proteins. Further studies are necessary to explore their roles in myopia.

## Discussion

In this study, we analyzed the exosomal protein content from the AH of the high myopia and control by LC-MS/MS. By analyzing the exosomal protein profile, we found that two proteins, APOA1 and OPTC, were common in the AH exosomes and could be potential exosomal protein markers for AH. Comparing the protein profile between myopia and control groups, 25 proteins showed significant enrichment in myopia patients. Two of these myopia-specific exosomal proteins, TTR and HPX have been found in previous myopia associated research. Further studies exploring their role in the development of myopia, would be beneficial. To our knowledge, we are the first to analyze the individual exosomal protein profile of the AH.

We found two proteins that were commonly had high expression in both myopia and control groups. APOA1 encodes apolipoprotein A I, which is the major protein component of high-density lipoprotein (HDL) in the plasma 36. APOA1 protein has been found in exosomes derived from the saliva, urine, platelets, and hepatocytes 30, 31, 37, 38. OPTC is a member of the small leucine-rich repeat protein (SLRP) family and is present in various eye tissues such as vitreous, cornea, iris, ciliary body, optic nerve, choroid, and retina 32. There are no previous reports showing OPTC as an exosomal protein. We suggest OPTC could be an AH-specific exosomal marker.

By comparing the exosomal protein profile between myopia and control groups, we identified 25 myopia-specific exosomal proteins (Table 3). In previous studies, Shao et al. showed that the expression of HPX and TTR in serum were positively correlated with myopia patients 33. Higher TTR levels have also been detected in the vitreous of myopia, macular detachment, and macular hole patients 39. Recently, exosomes have been indicated to pass through the blood-brain barrier 40, it would be possible for the AH exosomes carrying HPX or TTR to also pass through the blood-retinal barrier. In the eye development of zebrafish model, the expression of DKK3 was suggested to be associated with eye size 41.

Our results provide pioneer findings for the exploration of the exosomal protein profile in myopia development. Further studies identifying the target tissues of these exosomes and understanding the function of these myopia-specific proteins may provide significant information for the diagnosis, clinical treatment, and prognosis of myopia.

## Figures and Tables

**Table 1:** Information of patients with and without myopia

Group	Number	Gender	Age	Axial length (mm)
Myopia	M1	M	65	27.27
	M2	M	73	27.56
	M3	M	60	28.26
	M4	F	64	30.05
	M5	M	49	27.80
	M6	F	60	27.27
	M7	M	73	27.56
	M8	M	59	28.64
	M9	M	68	26.78
			63.44 ± 7.57	**27.91 ± 0.97**
Control	N1	F	53	23.18
	N2	F	77	23.10
	N3	M	71	24.96
	N4	F	61	22.79
	N5	M	86	25.59
	N6	F	90	23.31
	N7	M	58	23.30
	N8	M	72	23.29
	N9	F	82	22.34
			72.22 ± 12.86	**23.54 ± 1.04**

**Table 2:** Concentration of exosomes in AH

		10^5^ particles/μl
Myopia	M1	2.35
	M2	1.87
	M3	3.25
	M4	2.83
	M5	2.03
	M6	3.66
	M7	2.3
	M8	8.48
	M9	13.77
	**AVE.**	**4.29±4.01**
Control	N1	0.3
	N2	7.7
	N3	4.6
	N4	3.36
	N5	5.76
	N6	0.82
	N7	2.47
	N8	4.45
	N9	7.83
	**AVE.**	**4.14±2.87**

**Table 3:** Significantly changed exosomal proteins in myopia patients

Protein IDs	Protein Name	Gene Name	Control	Myopia	*p*-value
			Mean NSC ± SD	Mean NSC ± SD	
P02790	Hemopexin	HPX	0 ± 0	1.039 ± 1.213	3.32E-02
P02749	Beta-2-glycoprotein 1	APOH	0.202 ± 0.401	1.464 ± 1.525	3.96E-02
P06727	Apolipoprotein A-IV	APOA4	0.305 ± 0.458	1.908 ± 1.346	7.17E-03
B4DPN0	cDNA FLJ51265		0.202 ± 0.401	1.137 ± 1.134	4.20E-02
B4E1B2	cDNA FLJ53691		0.617 ± 1.546	3.384 ± 3.434	4.94E-02
P04196	Histidine-rich glycoprotein	HRG	0.31 ± 0.465	1.506 ± 1.254	2.26E-02
B2R8I2	cDNA FLJ93914		0.31 ± 0.465	1.506 ± 1.254	2.26E-02
A6XMH1	Transthyretin	TTR	2.662 ± 2.503	9.51 ± 8.2	3.88E-02
A0A087WV45	Transthyretin	TTR	2.662 ± 2.503	9.51 ± 8.2	3.88E-02
A0A087WT59	Transthyretin	TTR	3.618 ± 2.145	10.355 ± 7.589	2.99E-02
E9KL36	Transthyretin	TTR	4.641 ± 2.917	13.16 ± 9.822	3.31E-02
P02766	Transthyretin	TTR	4.641 ± 2.917	13.16 ± 9.822	3.31E-02
A6XGL1	Transthyretin	TTR	4.641 ± 2.917	13.16 ± 9.822	3.31E-02
Q6DHW4	Uncharacterized protein		0.93 ± 0.819	2.084 ± 0.875	1.07E-02
Q5CZ94	DKFZp781M0386		0.93 ± 0.819	2.084 ± 0.875	1.07E-02
Q6PJG0	Uncharacterized protein		0.93 ± 0.819	2.084 ± 0.875	1.07E-02
Q6P2J1	Uncharacterized protein		0.93 ± 0.819	2.084 ± 0.875	1.07E-02
Q7Z2U7	Uncharacterized protein		0.93 ± 0.819	2.084 ± 0.875	1.07E-02
Q6GMV8	Uncharacterized protein		0.93 ± 0.819	2.084 ± 0.875	1.07E-02
A0A5E4	Uncharacterized protein		0.93 ± 0.819	2.084 ± 0.875	1.07E-02
Q8NEJ1	Uncharacterized protein		0.93 ± 0.819	2.084 ± 0.875	1.07E-02
Q6P5S3	Uncharacterized protein		0.93 ± 0.819	2.084 ± 0.875	1.07E-02
P02652	Apolipoprotein A-II	APOA2	0.924 ± 0.93	2.046 ± 0.754	1.29E-02
V9GYM3	Apolipoprotein A-II	APOA2	0.924 ± 0.93	2.046 ± 0.754	1.29E-02
O43532	RIG-like 7-1		1.562 ± 0.668	2.88 ± 1.548	3.90E-02
Q8N294	cDNA FLJ33633		1.562 ± 0.668	2.88 ± 1.548	3.90E-02
B3KS70	cDNA FLJ35660		1.562 ± 0.668	2.88 ± 1.548	3.90E-02
B4DI69	cDNA FLJ59893		1.562 ± 0.668	2.88 ± 1.548	3.90E-02
Q9UBP4	Dickkopf-related protein 3	DKK3	1.562 ± 0.668	2.88 ± 1.548	3.90E-02
F6SYF8	Dickkopf-related protein 3	DKK3	1.562 ± 0.668	2.88 ± 1.548	3.90E-02
B4DID6	cDNA FLJ52545		1.562 ± 0.668	2.88 ± 1.548	3.90E-02
P01034	cystain-C	CST3	3.116 ± 1.381	5.661 ± 3.109	4.63E-02
A0A0K0K1J1	cystatin C	CST3	3.116 ± 1.381	5.661 ± 3.109	4.63E-02

#NSC: Normalized spectral count.

## Abbreviations

AH: aqueous humor
APOA1: apolipoprotein A1
HPX: hemopexin
LC-MS/MS: liquid chromatography-tandem mass spectrometry
OPTC: opticin
PCA: principal component analysis
TTR: transthyretin

## References

[1] Wu P.C (2019). Update in myopia and treatment strategy of atropine use in myopia control. Eye (Lond).

[2] Morgan I.G, K (2012). Ohno-Matsui, and S.M. Saw, Myopia. Lancet.

[3] Cooper J (2018). and A.V. Tkatchenko, A Review of Current Concepts of the Etiology and Treatment of Myopia. Eye Contact Lens.

[4] Kang P, Optical pharmacological strategies of myopia control Clin Exp Optom. 2018; 101(3): 321-332.

[5] Pan C.W, D (2012). Ramamurthy, and S.M. Saw, Worldwide prevalence and risk factors for myopia. Ophthalmic Physiol Opt.

[6] Lin L.L (2004). Prevalence of myopia in Taiwanese schoolchildren: 1983 to 2000. Ann Acad Med Singapore.

[7] Guo Y.H (2012). Self-reported myopia in Taiwan: 2005 Taiwan National Health Interview Survey. Eye (Lond).

[8] Russo A (2014). Myopia onset and progression: can it be prevented?. Int Ophthalmol.

[9] Goel M (2010). Aqueous humor dynamics: a review. Open Ophthalmol J.

[10] Huang A.S, B.A (2018). Francis, and R.N. Weinreb, Structural and functional imaging of aqueous humour outflow: a review. Clin Exp Ophthalmol.

[11] Schlotzer-Schrehardt U (2003). Matrix metalloproteinases and their inhibitors in aqueous humor of patients with pseudoexfoliation syndrome/glaucoma and primary open-angle glaucoma. Invest Ophthalmol Vis Sci.

[12] Klenkler B (2004). and H. Sheardown, Growth factors in the anterior segment: role in tissue maintenance, wound healing and ocular pathology. Exp Eye Res.

[13] Maatta M (2005). Matrix metalloproteinases and their tissue inhibitors in aqueous humor of patients with primary open-angle glaucoma, exfoliation syndrome, and exfoliation glaucoma. J Glaucoma.

[14] Jia Y, D.N (2014). Hu, and J. Zhou, Human aqueous humor levels of TGF- beta2: relationship with axial length. Biomed Res Int.

[15] Jia Y (2014). MMP-2, MMP-3, TIMP-1, TIMP-2, and TIMP-3 protein levels in human aqueous humor: relationship with axial length. Invest Ophthalmol Vis Sci.

[16] Zhang Y (2018). Expression of cytokines in aqueous humor from fungal keratitis patients. BMC Ophthalmol.

[17] Suzuki N (2018). Cytokine levels in the aqueous humor are associated with corneal thickness in eyes with bullous keratopathy. Am J Ophthalmol.

[18] Zhang K (2016). Elevated Transforming Growth Factor-beta2 in the Aqueous Humor: A Possible Explanation for High Rate of Capsular Contraction Syndrome in High Myopia. J Ophthalmol.

[19] Zhu X.J (2016). Elevated TGF-beta2 level in aqueous humor of cataract patients with high myopia: Potential risk factor for capsule contraction syndrome. J Cataract Refract Surg.

[20] Raposo G (2013). and W. Stoorvogel, Extracellular vesicles: exosomes, microvesicles, and friends. J Cell Biol.

[21] Colombo M, G (2014). Raposo, and C. Thery, Biogenesis, secretion, and intercellular interactions of exosomes and other extracellular vesicles. Annu Rev Cell Dev Biol.

[22] Mei F (2017). Potentially Important MicroRNAs in Form-Deprivation Myopia Revealed by Bioinformatics Analysis of MicroRNA Profiling. Ophthalmic Res.

[23] Wecker T (2016). MicroRNA Profiling in Aqueous Humor of Individual Human Eyes by Next-Generation Sequencing. Invest Ophthalmol Vis Sci.

[24] Dismuke W.M (2015). Human aqueous humor exosomes. Exp Eye Res.

[25] Kang G.Y (2014). Exosomal proteins in the aqueous humor as novel biomarkers in patients with neovascular age-related macular degeneration. J Proteome Res.

[26] Hoffman E.A (2009). Regulation of myocilin-associated exosome release from human trabecular meshwork cells. Invest Ophthalmol Vis Sci.

[27] Stamer W.D (2011). Protein profile of exosomes from trabecular meshwork cells. J Proteomics.

[28] Drewry M.D (2018). Differentially expressed microRNAs in the aqueous humor of patients with exfoliation glaucoma or primary open-angle glaucoma. Hum Mol Genet.

[29] Chen C.F (2019). Expression Profiling of Exosomal miRNAs Derived from the Aqueous Humor of Myopia Patients. Tohoku J Exp Med.

[30] Gonzalez-Begne M (2009). Proteomic analysis of human parotid gland exosomes by multidimensional protein identification technology (MudPIT). J Proteome Res.

[31] Gonzales P.A (2009). Large-scale proteomics and phosphoproteomics of urinary exosomes. J Am Soc Nephrol.

[32] Friedman J.S (2002). Protein localization in the human eye and genetic screen of opticin. Hum Mol Genet.

[33] Shao J (2012). [Proteomics analysis of serum biomarkers in patients with pathological myopia]. Zhonghua Yan Ke Za Zhi.

[34] Shao J (2011). Vitreous and serum levels of transthyretin (TTR) in high myopia patients are correlated with ocular pathologies. Clin Biochem.

[35] Shao J (2013). Functional analysis of misfolded transthyretin extracted from abnormal vitreous with high myopia related ocular pathologies. Clin Chim Acta.

[36] van der Vorst, E.P.C, High-Density Lipoproteins, Apolipoprotein A1 Subcell Biochem. 2020; 94: 399-420.

[37] Pienimaeki-Roemer A (2015). Lipidomic and proteomic characterization of platelet extracellular vesicle subfractions from senescent platelets. Transfusion.

[38] He M (2015). Hepatocellular carcinoma-derived exosomes promote motility of immortalized hepatocyte through transfer of oncogenic proteins and RNAs. Carcinogenesis.

[39] Shao J, Y (2011). Xin, and Y. Yao, Correlation of misfolded transthyretin in abnormal vitreous and high myopia related ocular pathologies. Clin Chim Acta.

[40] Banks W.A (2020). Transport of Extracellular Vesicles across the Blood-Brain Barrier: Brain Pharmacokinetics and Effects of Inflammation. Int J Mol Sci.

[41] Chiang C.Y (2020). Novel eye genes systematically discovered through an integrated analysis of mouse transcriptomes and phenome. Comput Struct Biotechnol J.

